# Eye tracking reveals expert gaze patterns during intracytoplasmic sperm injection

**DOI:** 10.1038/s41598-026-57054-8

**Published:** 2026-06-19

**Authors:** Azusa Asai, Hayato Hagiwara, Kazuki Hano, Tsubasa Kasui, Tatsuya Yoshimi, Masaki Takasu, Tadayoshi Aoyama, Kenji Kato

**Affiliations:** 1 Laboratory for Clinical Evaluation with Robotics, Assistive Robot Center, National Center for Geriatrics and Gerontology, Obu, Aichi 474-8511 Japan; 2 Department of Mechanical Systems Engineering, Nagoya University, Nagoya, Aichi 464-8603 Japan; 3 Center for One Medicine Innovative Translational Research (COMIT), Institute for Advanced Study, Gifu University, Gifu, Gifu 501-1193 Japan; 4 Nishitan ART Clinic Nagoya-ekimae, Nagoya, Aichi 450-6408 Japan; 5 Department of Mechatronics Engineering, Faculty of Science and Technology, Meijo University, Nagoya, Aichi 468-8502 Japan

**Keywords:** Medical research, Neuroscience

## Abstract

Intracytoplasmic sperm injection (ICSI) is a highly complex procedure that involves injecting a single sperm into an oocyte, requiring extensive training and advanced technical expertise, making it a task performed by specialists. Acquiring specialized microinjection skills alone often requires several years of training. ICSI performance has been primarily evaluated based on developmental outcomes, with little detailed assessment of operators’ intrinsic skills. We analyzed expertise during microinjection using eye-tracking technology, which was recently employed in surgical and sports domains to evaluate expert performance. Eye tracking was used to compare the fixation patterns and eye-gaze behaviors of experts and novices during microinjection. Our results showed that the experts had shorter and more consistent procedural times than the novices did. In contrast, the novices initially took longer times; although their time gradually decreased, they remained unstable. This difference was particularly noticeable during oocyte rotation. Similar patterns were observed for fixation duration and the number of saccades. The heat maps and gaze plots revealed interesting distinctions between experts and novices. The experts exhibited efficient and highly consistent eye gaze patterns. Their eye-gaze data may contribute to developing AI-driven automated ICSI skill evaluation systems and AI- and robotics-based ICSI technical support and automation methods.

## Introduction

Highly skilled manual operations often rely on *tacit knowledge*, a form of expertise that is difficult to verbalize yet essential for refined motor performance. Polanyi described this as follows: “We can know more than we can tell,” highlighting the existence of implicit cognitive and motor strategies that underpin expert behaviors^1^. Experts, when performing fine manipulation tasks, seamlessly coordinate perception and action, synchronizing gaze and hand movements to anticipate and control delicate motions. From a neuroscientific perspective, this coupling is supported by the two-stream hypotheses of visual processing^2^ and internal forward models of predictive motor control^3^. Based on these frameworks, gaze does not merely react to movement but also serves as a predictive guide for planning and executing precise actions. Given the significant impact of ICSI success rates on clinical outcomes, the standardization of technical skill is a critical challenge in modern reproductive medicine. However, the reliance on individual experience often leads to technical disparities among embryologists. Thus, eye movements provide a unique insight into the cognitive and perceptual processes underlying skilled performance. Studies of natural behavior have shown that gaze is organized in a task-driven, goal-oriented manner^4,5^. In addition, expert performers exhibit anticipatory gaze patterns preceding hand actions^6,7^, introducing the concept of the *Quiet Eye*—a final, stable fixation immediately before a critical movement— and demonstrating that experts allocate attention more efficiently and with greater temporal stability than novices do. Similar findings have been reported in surgical and medical training domains, where metrics such as fixation duration, gaze entropy, and saccade frequency serve as objective indicators of expertise^8–10^. In this context, ICSI is an ideal model for studying fine visually guided manipulation in a biomedical setting. Reports from the World Health Organization (WHO) show that infertility affects approximately one in six people of reproductive age worldwide during their lifetime^11^. Therefore, Assisted Reproductive Technology (ART) has become a key clinical field, with ICSI representing a sophisticated and frequently used in vitro fertilization (IVF) technique^12^. This procedure requires the direct microinjection of a single sperm into an oocyte, a process that demands exceptional precision and visual–motor coordination^13^. The operator must correctly align and stabilize the oocyte, puncture it with a micropipette at an optimal angle and depth, and inject the sperm without damaging the meiotic spindle. Even a small deviation can compromise fertilization or embryo development. Given these constraints, extensive training is required to master ICSI, and differences in operator proficiency significantly influence outcomes^14^. However, current assessments rely primarily on performance outcomes such as fertilization or cleavage rates, without capturing the underlying process-level differences in attention and movement strategies. This lack of process-oriented evaluation hinders the systematization of education and standardization of ICSI practices.

Recent advances in eye tracking technology enable fine-grained visualization of operators’ attention and cognitive strategies in real-time, particularly in fields such as surgery and sports^15–18^. Eye-tracking technology can be used to estimate the arrangement of visual attention and the flow of thought by capturing eye movements and fixation positions during a task in a time series. In ICSI, where precise visual–motor coordination is critical, eye tracking can reveal how experts allocate their visual attention, stabilize their gazes, and minimize unnecessary saccades during micromanipulation. In particular, accurate recognition and alignment of the polar body of the oocyte are crucial for determining the injection angle and avoiding mechanical damage^19^. However, detailed analyses comparing gaze behavior between expert and novice embryologists during injection procedures remain unclear.

The aim of this study was to compare the gaze behavior of expert and novice operators during ICSI using eye-tracking analysis, with the goal of identifying the cognitive and operational characteristics associated with proficiency. The microinjection process was divided into two stages: (1) oocyte rotation (STEP 1) and (2) oocyte puncture (STEP 2). By quantifying the fixation stability, saccade frequency, and gaze distribution at each step, we sought to extract behavioral patterns specific to experts and propose novel quantitative indicators for skill assessment. These insights may facilitate the modeling of expert skills, enhance training programs, and provide principles for AI- and robotics-based automation support systems.

Several attempts have been made to automate or assist some parts of the ICSI procedure using AI and robotics. There has been progress in sperm immobilization and injection automation^20,21^; however, oocyte rotation remains a particularly challenging task. Efforts include polar body detection-based automation^22^ and AI-guided manipulation learning using expert data^23^. These approaches have shown promise. However, they often lack the ’clinical adaptability’ required to handle the diverse and unpredictable nature of living cells, which experienced embryologists manage through intuitive judgment. By quantitatively characterizing the visual and motor strategies of experts, this study aims to establish a scientific foundation for skill evaluation. These insights provide not only a benchmark for objective assessment but also the essential parameters required for developing advanced training programs and AI-assisted support systems that mimic the decision-making processes of master embryologists.

## Methods

### Participants

Fifteen participants (10 men and 5 women) affiliated with Nagoya University, Gifu University, and Nishitan ART Clinic Nagoya-ekimae participated in the study. They were divided into two groups based on their experience with ICSI procedures.

- Expert group: seven participants (two embryologists, one faculty member, one researcher, and three students). They had > 1 year of experience with ICSI procedures, with an average of 7.2 years of experience. Among them, the two embryologists had > 2 years of clinical experience, having performed at least 100 clinical ICSI procedures with a maintenance of 75–85% fertilization rate. The other experts (faculty member, researcher, and students) were categorized based on their technical proficiency in simulated environments, which was confirmed to be equivalent to the skill level of the embryologists.
- Novice group: Eight participants (one office worker and seven students). These participants had no prior experience with ICSI procedures. The age range of all participants was 20–43 years (Table 1).

E1, E3, and N3 were co-authors, but they were not informed of the evaluation categories at the time of measurement (Table 1).

**Table 1:** Subject Information.

Subject	Age	Gender	Dominant hand	Specialty	Position	Years
1	29	Male	Right	Expert	Researcher	8
2	23	Male	Right	Expert	Undergraduate student	3
3	35	Male	Right	Expert	Embryologist	12
4	26	Male	Right	Expert	Undergraduate student	3
5	23	Male	Right	Expert	Doctoral student	1.5
6	43	Male	Right	Expert	Assistant Professor	20
7	32	Male	Right	Expert	Embryologist	3
8	23	Male	Right	Novice	Postgraduate student	0
9	23	Male	Right	Novice	Postgraduate student	0
10	25	Female	Right	Novice	Academic Staff	0
11	25	Male	Right	Novice	Undergraduate student	0
12	21	Female	Right	Novice	Undergraduate student	0
13	21	Female	Right	Novice	Undergraduate student	0
14	20	Female	Right	Novice	Undergraduate student	0
15	22	Female	Right	Novice	Undergraduate student	0

**Fig. 1:**
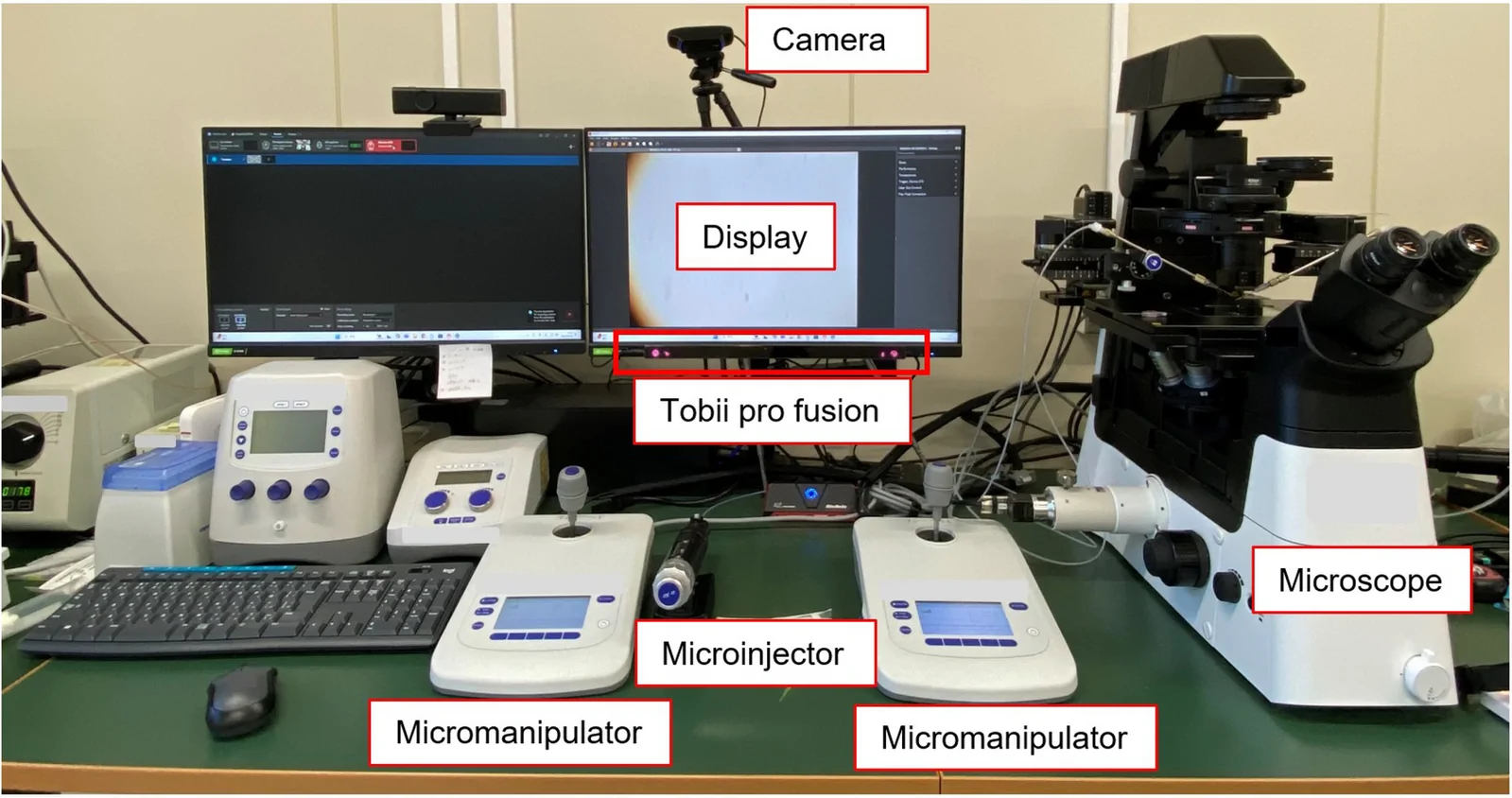
Overview of the experimental apparatus. A Tobii Pro Fusion eye tracker was mounted at the lower edge of the monitor. Both experts and novices performed ICSI procedures while observing the monitor.

### Eye tracking device

Eye-tracking data were obtained using the Tobii Pro Fusion (Tobii Technology, Stockholm, Sweden). The device is a remote eye tracker attached to a monitor, providing highly accurate eye tracking at a sampling rate of 120 Hz. Microscopic images during microinjection were displayed on a monitor, and an eye tracker was attached to the monitor to collect eye-tracking data (Fig. 1). Data were analyzed using the Tobii Pro Lab software.

### Oocyte preparation

Porcine ovaries were obtained from a local slaughterhouse (Gifu City Local Wholesale Market, Gifu, Japan), which provided permission for the use of unfertilized oocytes. Oocytes were collected using a porcine in vitro fertilized oocyte production medium kit (Research Institute for Functional Peptide, Yamagata, Japan; IFP101PEP-CK). Briefly, slaughterhouse-derived porcine ovaries were washed with saline, and cumulus cell complexes were isolated from follicles. Oocytes selected using a stereomicroscope were cultured. After maturation, cumulus cells were detached using 0.1% (w/v) hyaluronidase (SIGMA, H3884-100MG, Merck, Darmstadt, Germany), and oocytes that released the first polar body were selected and used in the experiment.

### Procedures

The oocytes were placed in a fixed position using a holding pipette. The time at which the micropipette started moving was indicated as the start of the operation, and the end of the operation was defined as the time at which the micropipette was completely withdrawn from the oocyte. For the microinjection procedure, sperm were replaced with ultrapure water, as oocyte development was not assessed. Each participant performed 10 consecutive ICSI microinjection manipulations, and corresponding eye-tracking data were obtained for each participant.

### Definition of the ICSI process

The ICSI manipulation process was divided into the following two steps.

- Total: the entire procedure from the start of the ICSI procedure to the end.
- STEP 1 (oocyte rotation): rotation of the oocyte and alignment of the polar bodies in the 12 or 6 o’clock direction.
- STEP 2 (puncture): injection of sperm into the oocyte using a micropipette.

We simultaneously observed eye-tracking and overall manipulation to compare the skills of the expert and novice participants.

### Eye tracking analysis

The acquired eye-tracking data were analyzed using Tobii Pro Lab software (version 24.21.435). The following metrics were quantified and compared between the expert and novice groups.

- Fixation: gaze remaining at a specific point for at least 60 ms.We utilized the Tobii I-VT Filter (Fixation) with a velocity threshold of 30°/s.
- Saccade: rapid movement of the gaze from one point to another. We utilized the Tobii I-VT Filter (Fixation) with a velocity threshold of 30°/s.
- Heat map: Spatial distribution of visual attention based on fixation duration.
- Gaze plot: analysis of the gaze trajectory showing fixation positions and their sequence.
- Areas of Interest (AOI): region of interest (ROI). We set an ROI on the polar body. Specifically, the Polar Body ROI was defined around the visually identified polar body for each trial. The size of the ROI was kept consistent across all trials.

For each operation, the procedure time (Total), time required for STEP 1 and STEP 2, number of fixations, average fixation duration, and number of saccades were calculated and compared between groups. Furthermore, the semantic interpretation of gaze was facilitated by the synchronized display of video footage and gaze plots during ICSI. A Dynamic AOI was established for the polar body in Tobii Pro Lab to analyze the duration time percentages were subsequently compared. Data represent the mean value±SEM (M±SEM) in each group. The difference in means between the expert and novice groups was analyzed using an unpaired Student’s t-test. Statistical significance was set at *p* < 0.01.

**Fig. 2:**
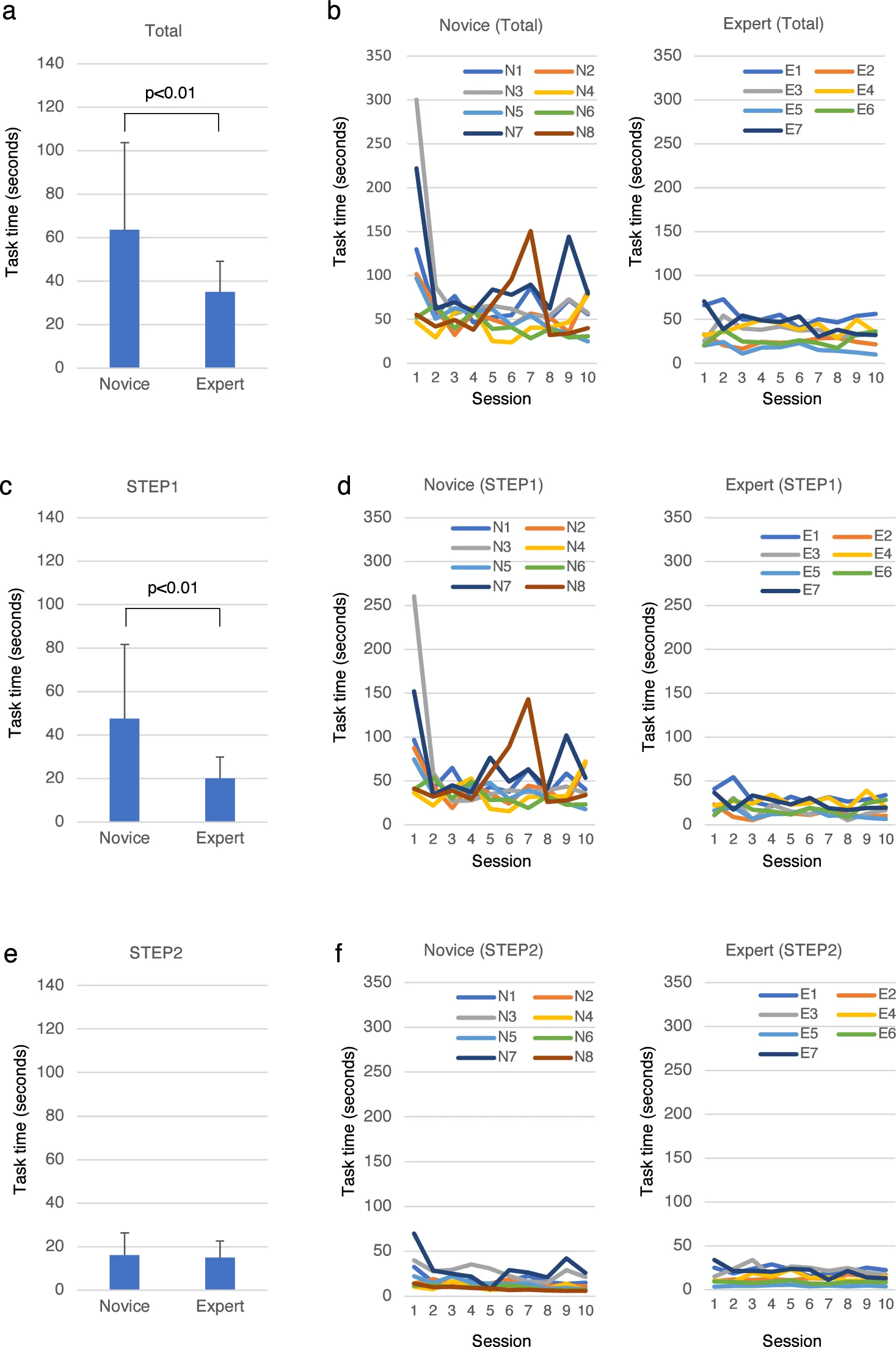
Task completion times. Comparison of task durations (M±SEM) for Total (**a** and **b**), STEP1 (**c** and **d**), and STEP 2 (**e** and **f**) between novices and experts during ICSI. Total, the entire procedure; STEP 1, oocyte rotation; STEP 2, puncture.

**Fig. 3:**
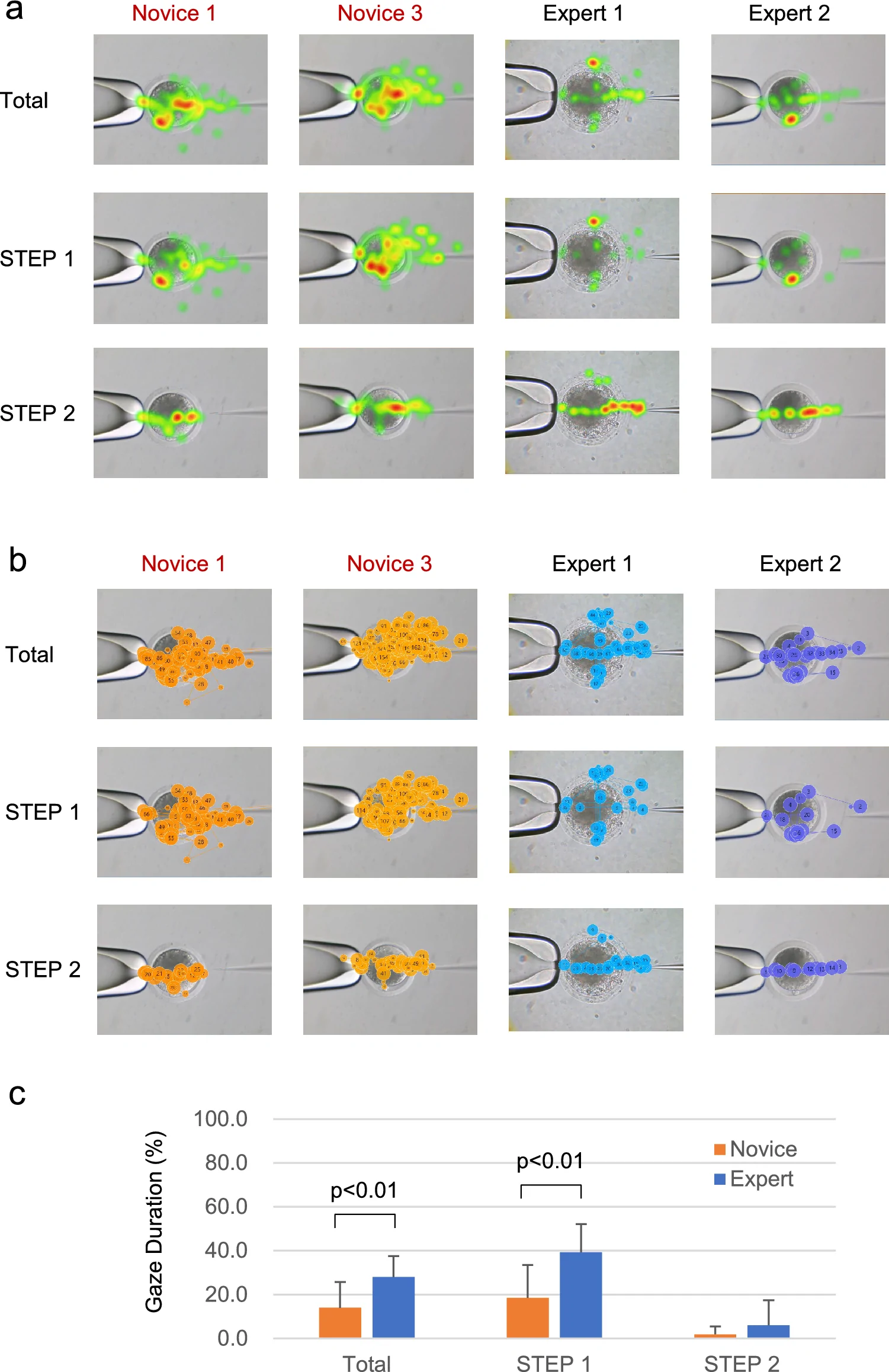
Visual representation and quantitative analysis of gaze behavior. (**a**) Heat maps, (**b**) gaze plots, and (**c**) comparison of gaze duration percentages between novices and experts during different task stages (Total procedure, STEP 1: oocyte rotation, and STEP 2: puncture). The gaze duration in c was calculated based on the ROI defined for the polar body. The bar graph in c presents the mean gaze duration (M ± SEM) for each stage, with significant differences indicated (*p* < 0.01). Representative gaze examples were selected from median performers in each group to ensure that they represent the typical patterns observed in the study.

**Fig. 4:**
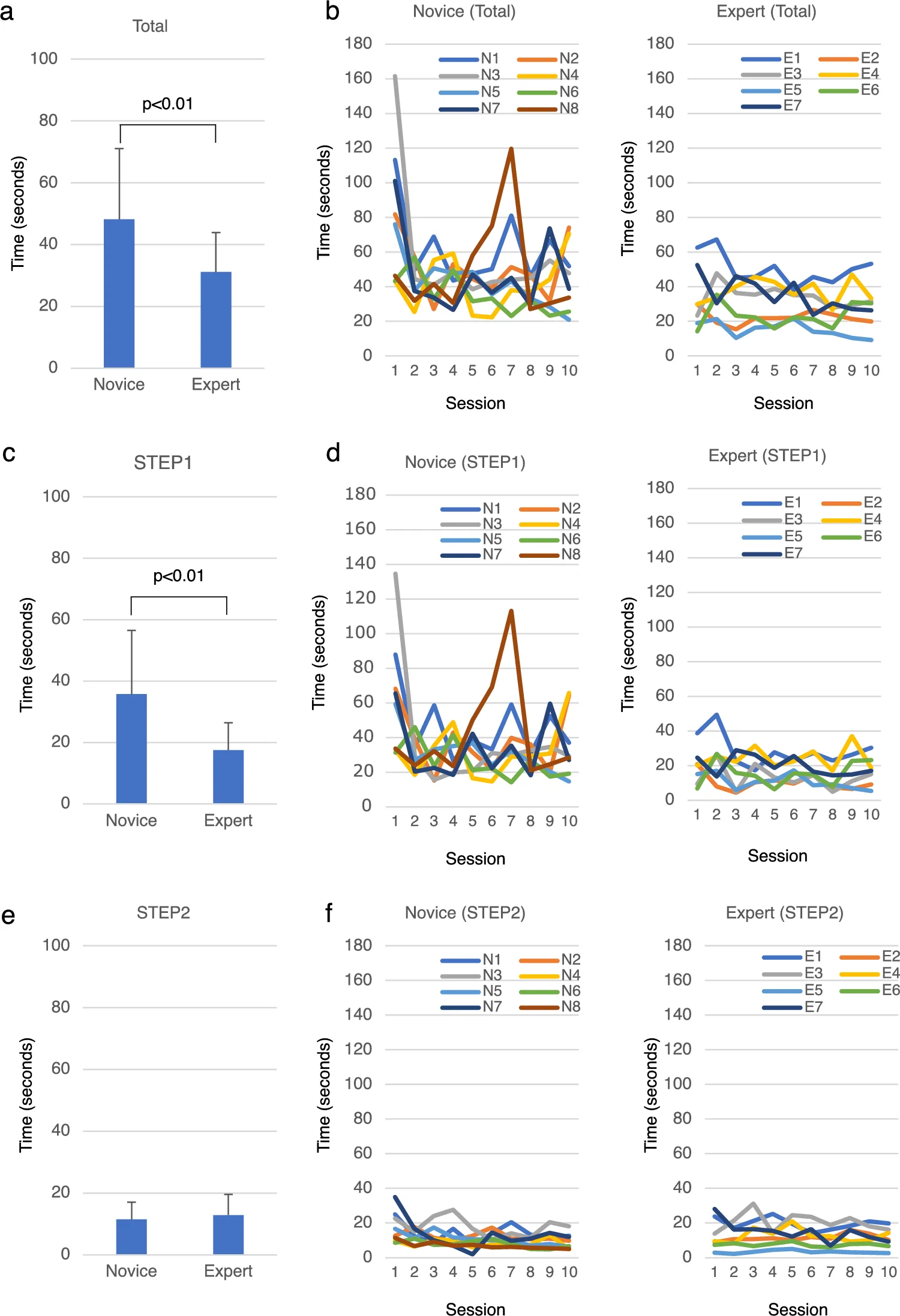
Comparison of fixation durations. Comparison of fixation durations (M±SEM) for Total (**a** and **b**), STEP 1 (**c** and **d**), and STEP 2 (**e** and **f**).

**Fig. 5:**
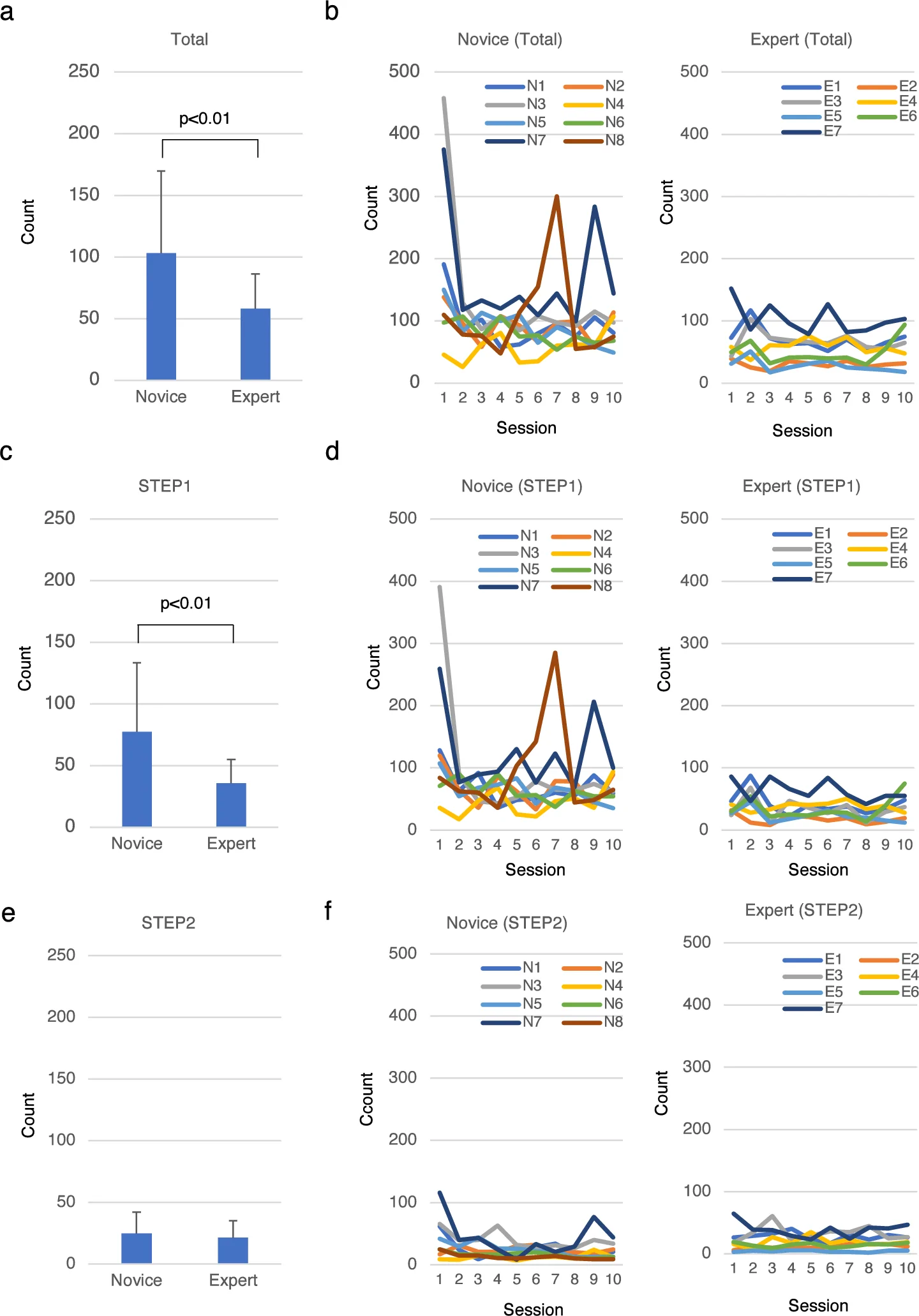
Comparison of number of fixations. Comparison of the number of fixations (M±SEM) for Total (**a** and **b**), STEP 1 (**c** and **d**), and STEP 2 (**e** and **f**).

**Fig. 6:**
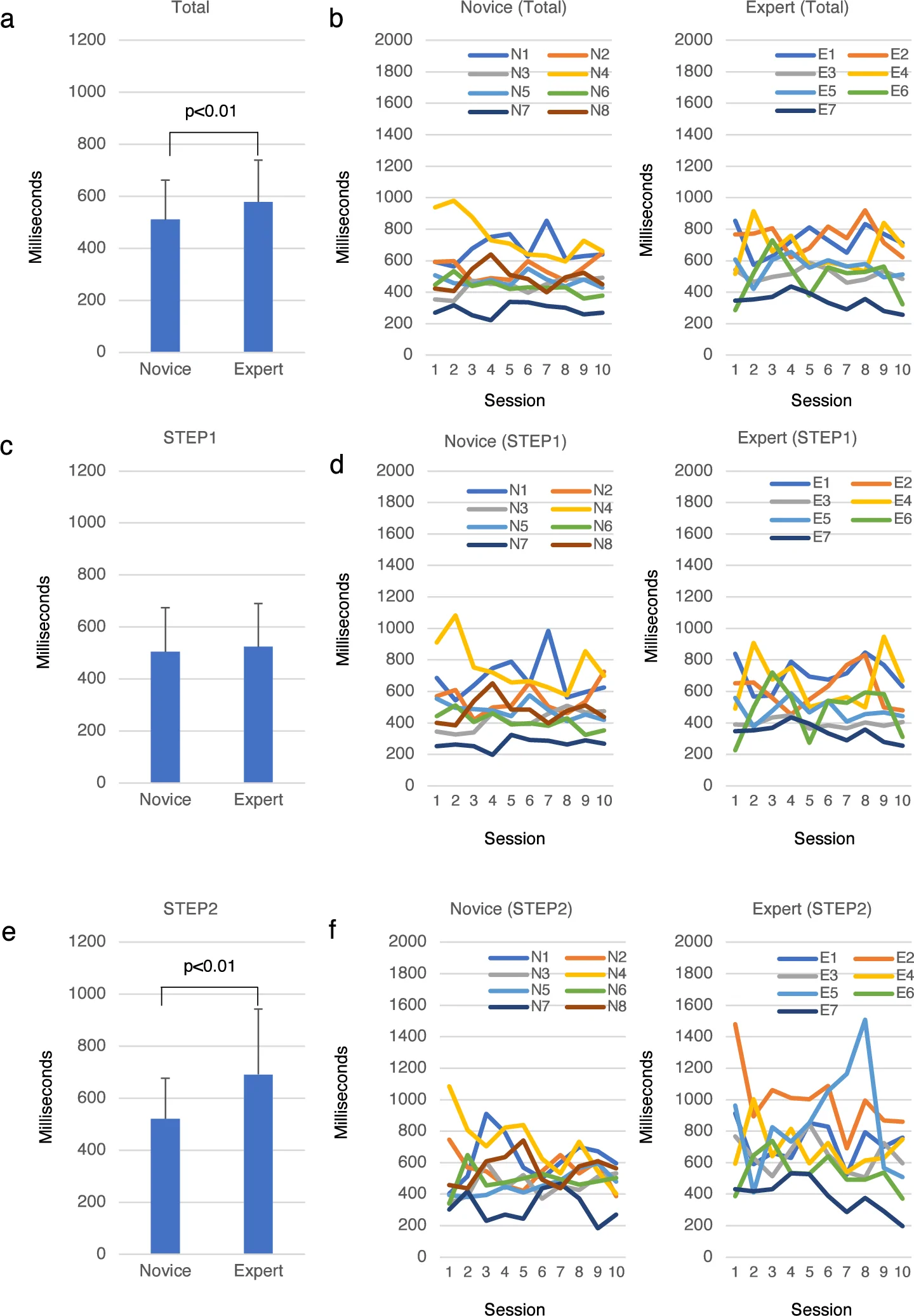
Comparison of the average fixation duration. Comparison of the average fixation duration for Total (**a** and **b**), STEP 1 (**c** and **d**), and STEP 2 (**e** and **f**).

**Fig. 7:**
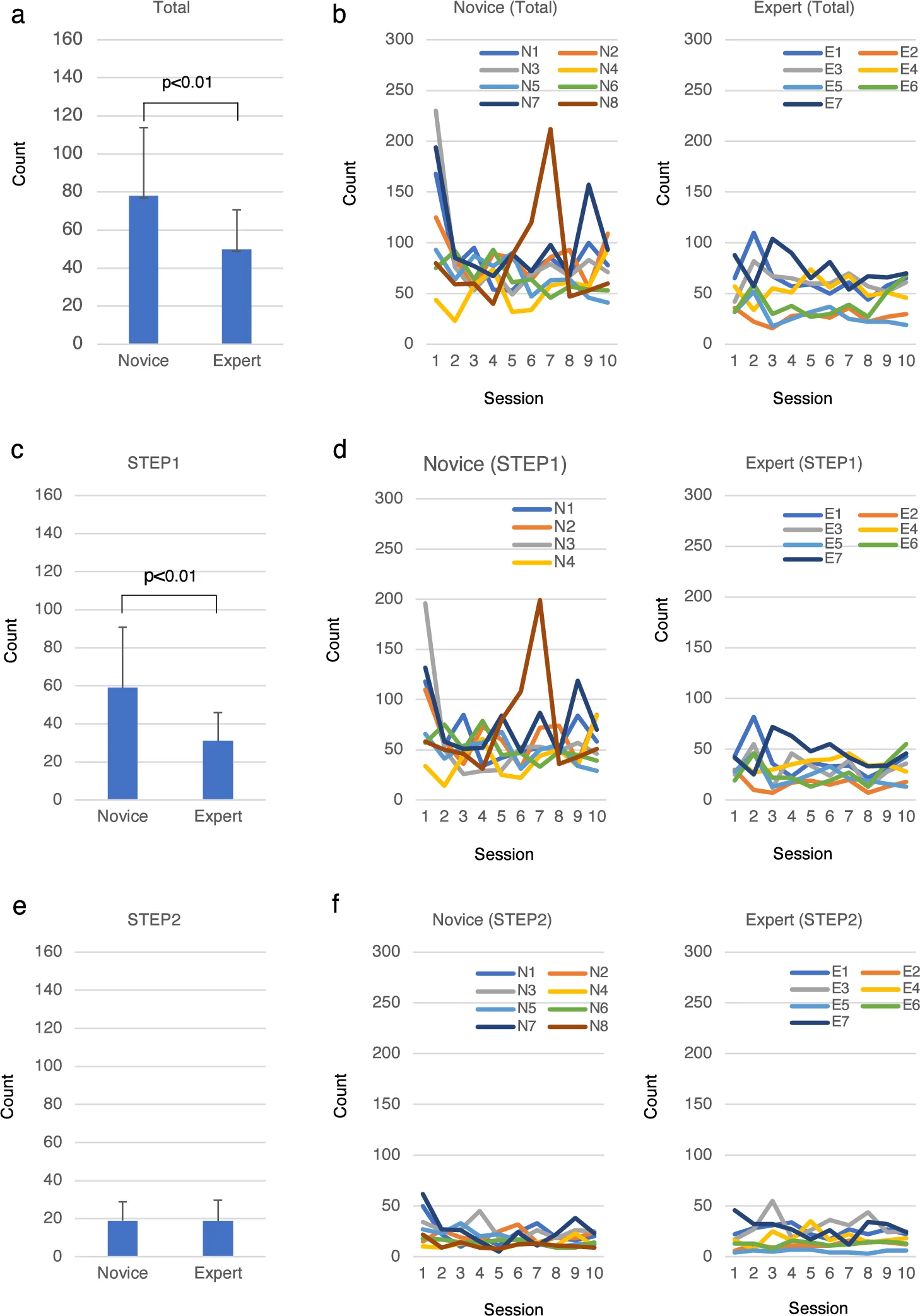
Comparison of the number of saccades. Comparing the number of saccades (M±SEM) for Total (**a** and **b**), STEP 1 (**c** and **d**), and STEP 2 (**e** and **f**).

## Results

### Task performance

In general, the operation time of surgical tasks was shorter for experts than for novices. For a successful ICSI procedure, it is important to quickly and accurately perform microinjections. Therefore, we first compared the duration of microinjections between the experts and novices. Each microinjection procedure was performed 10 times, and the task durations were recorded and compared.

The results clearly showed that the mean duration of microinjection was significantly shorter for experts than for novices (*p<0.01*) (Fig. 2a). The experts, from their first attempt, exhibited a high performance, characterized by consistently short and stable task completion times (Fig. 2b, right). In contrast, novices recorded the longest duration on the first attempt, followed by gradual reductions with repeated trials. A learning effect was evident, as all novices achieved task times below the novice average by the third attempt; however, some novices showed variability and inconsistency in subsequent trials (Fig. 2b, left).

The microinjection procedure was divided into two steps: oocyte rotation (STEP 1) and puncture (STEP 2). We compared the time spent by the experts and novices on each step. The results showed a significant difference in the time spent on STEP 1 between novices and experts (Fig. 2c,d). However, no difference was observed in the time taken for STEP 2 (Fig. 2e,f). These results indicate that experts perform oocyte rotation (STEP 1) more efficiently than novices.

### Gaze behavior analysis

Figure 3a shows heat maps illustrating the gaze patterns of the experts and novices during microinjection. The heat maps represent the location and duration of fixation, with red areas indicating longer fixation times and yellow to green areas indicating shorter fixation times. The heat maps revealed that the experts’ gazes were predominantly fixed on the polar body with coordinated horizontal and vertical movements. However, the gaze pattern for novices was irregular and dispersed, often focusing on the needle tip.

When analyzed separately, experts’ fixations during STEP 1 (rotation) were primarily centered on the polar body, whereas novices’ fixations were widely distributed. During STEP 2 (puncture), no notable differences were found between the groups; both groups primarily gazed horizontally near the needle position. These findings suggest that gaze behavior differs between experts and novices primarily during oocyte rotation in STEP 1, and that experts tend to maintain visual focus on the polar body throughout ICSI manipulation.

Gaze plots (Fig. 3b) show the sequence and distribution of the fixations. These results are consistent with the heatmap analysis shown in Fig. 3a. The experts initially scanned the whole area by moving their eyes horizontally and vertically, and subsequently focused on the polar body after finding it. Most fixations were inside the oocyte and were few in number. In contrast, the novice fixated both inside and outside the oocyte, with a more scattered pattern and less consistent sequence. Novices also had a higher total number of fixations than experts did.

Furthermore, the gaze plots for each STEP were compared. The results showed that in the STEP 1 gaze plot, experts concentrated their fixations on the polar body after examining the entire oocyte and identifying it. In contrast, the novices’ fixations were more scattered and extended outside the oocyte. Next, in the gaze plot in STEP 2, after the expert had initially identified the holding pipette, the fixations were found to be almost horizontal and concentrated, especially on the membrane on the puncture side. Novices, however, demonstrated horizontal but more dispersed and inconsistent fixation patterns.

In Fig. 3c, we set an ROI over the polar body to quantitatively compare the percentage of gaze duration between expert and novice operators. Our analysis revealed that experts allocated a significantly longer gaze duration time to the polar body during the oocyte rotation phase (Step 1; *p* < 0.01)

### Fixation metrics

Next, we compared fixation durations across the entire procedure (Total), STEP 1, and STEP 2. Experts showed significantly shorter fixation durations than did the novices during the total task and STEP 1 tasks (Fig. 4a–d), possibly reflecting a shorter overall task time. No significant differences were observed in STEP 2 (Fig. 4e,f).

We compared the number of fixations in Total, as well as in STEP 1 and STEP 2. The total number of fixations was lower for experts than for novices (Fig. 5a,b). Similarly, in STEP 1, the number of fixations was lower for experts than for novices (Fig. 5c,d). In STEP 2, although there was some variation, no difference in the number of fixations between experts and novices was observed (Fig. 5e,f).

Figure 6 shows the comparison of the average fixation duration for the total task as well as for STEP 1 and STEP 2. The average fixation duration across the total task was longer for experts than for novices (experts 578 ± 161 ms, novices 510 ± 153 ms, *p<0.01*) (Fig. 6a,b). This finding indicates that although the experts exhibited shorter total fixation durations (Fig. 4a), each fixation was longer and more deliberate, suggesting a higher level of visual concentration. In STEP 1, no significant difference was observed between experts and novices (experts 524 ± 165 ms, novices 504 ± 170 ms) (Fig. 6c,d). However, in STEP 2, experts demonstrated significantly longer average fixation durations than novices did (expert 691 ± 251 ms, novices 521 ± 156 ms, *p<0.01*) (Fig. 6e,f), suggesting that experts concentrate particularly during puncture. Combined with the heat map and gaze plot results, these findings indicate that expert fixation is mostly focused on the needle tip at the moment of puncture in STEP 2.

### Saccade analysis

The total number of saccades was significantly lower for the experts than for the novices (Fig. 7a,b). Similarly, in STEP 1, experts showed fewer saccades than novices did (Fig. 7c,d). However, no differences were observed in STEP 2 (Fig. 7e,f). These results indicate that experts performed ICSI with fewer unnecessary eye movements, particularly during oocyte rotation.

## Discussion

In this study, eye-gaze behavior and task performance of experts and novices during ICSI were quantitatively compared using an eye-tracking system. Our findings revealed that the experts were significantly faster than the novices at completing the microinjection task and showed stable performance from the first trial. Notably, distinct differences in visual attention were observed during the oocyte rotation phase (STEP 1): experts consistently gazed at the polar body with fewer and shorter fixations and fewer saccades, reflecting efficient visuomotor coordination. In contrast, the novices exhibited scattered gaze patterns and frequent fixations on the needle tip, indicating unstable attentional control. These findings reveal that expert-level proficiency in ICSI is characterized by a shorter task time and organized and economical visual search behavior. To our knowledge, this is the first study to quantitatively characterize expert-specific gaze allocation during ICSI-like microinjection tasks, particularly during oocyte rotation and polar body identification. Overall, our findings highlight the feasibility of using quantitative gaze metrics as assessment of technical skill proficiency and may serve as a foundation for developing advanced training programs and AI-assisted support systems.

Skill differences between the two groups were clarified. As indicated by the heat map results, the experts maintained a concentrated gaze on the polar body, exhibiting shorter fixation durations, fewer fixations, and reduced saccade frequency, as evidenced by a significantly higher percentage of duration within the Polar Body ROI compared to novices (Fig. 3c), demonstrating efficient visuomotor coordination reflected in their gaze patterns. Meanwhile, novices displayed widely dispersed gazes, frequently fixating on the needle tip and outside the oocyte, indicating attentional instability. These differences in eye-gaze behavior are similar to the eye-gaze characteristics reported by surgical and sports experts^9,16,24^, suggesting that eye-tracking metrics could serve as objective indicators for evaluating skill proficiency in ICSI procedures. However, it is crucial to distinguish the specific complexities of ICSI from those of macroscopic surgical tasks. Unlike macro-surgery, which often involves relatively stable anatomical targets, ICSI requires the high-speed, high-precision manipulation of living cells under high magnification. This environment demands a unique multitasking capability: operators must continuously monitor the dynamic physiological responses of the oocyte–such as cytoplasmic membrane stretching–while simultaneously executing sub-micron level adjustments. Our findings suggest that expert-level visual strategies are specifically optimized for this micro-scale multitasking, a dimension not fully captured in macro-surgical behavior analysis.

Differences in cognitive strategies were also examined. In this study, eye-gaze behavior showed prominent differences during STEP 1, whereas the two groups did not significantly differ during STEP 2. This finding suggests that visualization of the polar body and subsequent oocyte rotation are key elements of ICSI skill, and that this process is directly linked to the allocation of visual attention. This observation supports the idea that, in tasks requiring precise spatial recognition and judgment, experts process information with the minimum necessary eye movements^15,18^. Furthermore, the average fixation duration was longer in STEP 2. Gaze trajectories might appear similar; nevertheless, experts might acquire more information about the oocytes. Oocyte damage most frequently occurs during injection^19,25,26^. Previous studies have shown that oocytes that do not exhibit cytoplasmic membrane stretching during injection pipette puncture are unlikely to recover and have a higher probability of degeneration^27^. Moreover, experts may simultaneously assess the extent of oocyte stretching.

Notably, the concept of the Quiet Eye (QE) refers to “a sustained and spatially stable gaze fixation of at least 100 ms duration within 3° of visual angle on a critical location or object in the task environment”^28^. It represents the ability to fixate on the right place at the right time while performing critical actions. For instance, in precision movements such as basketball three-point shots and golf putting^29,30^, the QE observed in skilled athletes has recently garnered significant research attention^28^. It is a cognitive and psychological skill that enhances attention and concentration, thereby improving motor performance. QE Training (QET) can extend the duration of QE. By acquiring the eye-gaze behaviors of experts through the QET, it is possible to stabilize attentional control and motor planning. This method is reportedly effective for suppressing performance decline, even under psychological stress, and maintaining an optimal mind-body state^31^. Based on the data obtained in this study, it is expected that incorporating expert eye-gaze patterns into QET can optimize ICSI procedures.

Eye-tracking metrics such as fixation duration and number of saccades are well-established parameters that reflect cognitive load and concentration during complex tasks^9^. In this study, their contribution to the quantitative assessment of procedural stability and learning status was highlighted. Specifically, experts’ eye-gaze exhibited a clear “transition from exploration to concentration,” which was a critical difference compared to novices.

The findings of this study have significant educational implications. Currently, ICSI training primarily relies on on-the-job training based on empirical rules, with limited opportunities for quantitative evaluation of procedural skills or objective feedback^14^. By elucidating the eye-gaze strategies of experts through eye-tracking analysis, it is possible to visualize skill acquisition, specifically identifying where novices should focus their attention and in what sequence they should perform operations. This approach opens the possibility of designing real-time gaze-based feedback systems that enhance learning efficiency and facilitate skill transfer.

The mimicking of expert eye-gaze patterns is expected to enhance the precision of automated systems. Furthermore, the findings of this study can contribute to developing AI- and robotics-driven ICSI support technologies. Attempts to automate tasks such as sperm immobilization and injection using microrobots have been described in several reports^21,32^. However, for tasks requiring expert judgment, such as oocyte rotation and polar body detection, it is crucial to incorporate support algorithms that mimic human eye gaze and decision-making processes. The effectiveness of real-time assistive feedback systems using imitation learning was demonstrated in another study^23^. The eye-gaze patterns identified in this study provide significant insight into the design of such AI systems. Our research group at Nagoya University is leveraging these expert gaze metrics to develop AI-assisted support systems for ICSI^33^. We have recently implemented a real-time system that uses semantic segmentation to highlight the polar body, effectively reducing the perceptual burden on novices. A unique feature of our approach is the visualization of inference ’ambiguity,’ which fosters operator trust by intuitively communicating the system’s confidence. By offloading the cognitive task of polar body detection, this system alleviates the attentional bottleneck identified in our gaze analysis, allowing novice operators to focus on precise pipette manipulation . Thus, we aim to enable embryologists to achieve consistent and successful oocyte rotations through this integrated support.

This study has some limitations. It was conducted in a simulated environment using porcine oocytes, which differ from those in actual clinical settings. Additionally, we did not perform an integrated multimodal analysis that combined eye tracking data with other information, such as hand movements, surface electromyography, or subjective fatigue levels. Future research should focus on developing proficiency models that integrate eye-gaze data with multiple physiological and behavioral metrics.

The gaze data obtained from experts in this study provided valuable information regarding the allocation of visual attention. The finding suggests that presenting expert gaze patterns may provide a potential target for training interventions aimed at improving the operational stability of novices. In the future, we anticipate significantly enhancing the quality and efficiency of training by developing an AI model that integrates multiple elements, such as experts’ gazes, movements, and judgment timing, and subsequently implementing this model in training-support AI or ICSI simulators. Furthermore, establishing an objective skill assessment scale based on gaze information is expected to support the standardization and inheritance of ICSI skills, thereby contributing to the rectification of technical disparities in clinical settings.

## Data Availability

The data that support the findings of this study are available from the corresponding author upon reasonable request.
